# Intratumoral distribution of *EGFR* mutations and copy number in metastatic lung cancer, what impact on the initial molecular diagnosis?

**DOI:** 10.1186/1479-5876-12-131

**Published:** 2014-05-16

**Authors:** Audrey Mansuet-Lupo, Fouzia Zouiti, Marco Alifano, Anne Tallet, Marie-Christine Charpentier, Véronique Ducruit, Fabrice Devez, Fanny Lemaitre, Pierre Laurent-Puig, Diane Damotte, Hélène Blons

**Affiliations:** 1 UMRS 872, INSERM, Centre de Recherche des Cordeliers, Paris, France; 2 Université Paris Descartes, Sorbonne, Paris cité, France; 3 Université Pierre et Marie Curie, Paris, France; 4 Assistance Publique Hôpitaux de Paris, Department of Pathology, Hôpital Cochin-Hôtel Dieu, Paris, France; 5 Assistance Publique Hôpitaux de Paris, Department of Biology, Hôpital Européen Georges Pompidou, Paris, France; 6 Assistance Publique Hôpitaux de Paris, Department of Thoracic Surgery, Hôpital Cochin, Paris, France; 7 UMR-S775, INSERM, Centre Universitaire des Saints Pères, Paris, France; 8 INSERM, Université Paris Descartes, 45 Rue des Saints Pères, Paris 75006, France

**Keywords:** *EGFR* mutation, Genetic heterogeneity, *EGFR* amplification

## Abstract

**Background:**

Activating epidermal growth factor receptor (*EGFR*) mutations characterize a subgroup of non-small-cell lung cancer that benefit from first line EGFR tyrosine kinase inhibitors (EGFR-TKI). However, the existence of polyclonal cell populations may hinder personalized-medicine strategies as patients’ screening often depends upon a single tumor-biopsy sample. The purpose of this study is to clarify and to validate in clinical testing conditions the accuracy of *EGFR* genotyping using different tumor sites and various types of samples (transthoracic, surgical or endoscopic biopsies and cytology specimens).

**Methods:**

We conducted a retrospective review of 357 consecutive patients addressed for *EGFR* mutation screening in accordance with the directive of the European Medicines Agency (stage IV NSCLC). Fifty-seven samples were *EGFR* mutated and 40 had adequate tumor specimens for analysis on multiple spatially separated sites. Ten wild type samples were also analyzed. A total of 153 and 39 tumor fragments, from mutated and non-mutated cases respectively, were generated to analyze tumor heterogeneity or primary-metastatic discordances. After histological review of all fragments, *EGFR* genotyping was assessed using the routine diagnostic tools: fragment analysis for insertions and deletions and allele specific TaqMan probes for point mutations. *EGFR* copy number (CN) was evaluated by qPCR using TaqMan probes.

**Results:**

The identification of *EGFR* mutations was independent of localization within primary tumor, of specimen type and consistent between primary and metastases. At the opposite, for half of the samples, tumor loci showed different *EGFR* copy number that may affect mutation detection cut-off.

**Conclusions:**

This is the largest series reporting multiple *EGFR* testing in Caucasians. It validates the accuracy of *EGFR* mutation screening from single tumor-biopsy samples before first line EGFR-TKI. The unpredictable variability in *EGFR* CN and therefore in *EGFR* wild type/mutant allelic ratio justifies the implementation of sensitive methods to identify patients with *EGFR* mutated tumors.

## Introduction

Lung carcinoma is the first cause of death by cancer in the world, mostly because patients have an advanced stage disease at diagnosis [1]. Adenocarcinoma (ADC), the most frequent histological type is morphologically and biologically heterogeneous. Different architectural patterns have been described and different molecular pathways are involved in the carcinogenesis process [2]. Attention has largely been focused on proliferation pathways with the identification of mutations in oncogenes such as *KRAS*, *EGFR*, *ALK*, *HER2*, *PI3KCA* and *BRAF* that are potential or validated drug targets [3]. The first identified target in NSCLC was the EGF receptor. In 2004, *EGFR* activating mutations were identified in lung ADC and rapidly associated with response to EGFR-TKI [4,5]. Clinico-pathological features that correlate with these mutations include east-Asian ethnicity, adenocarcinoma histology, female sex, and never smoking history. In lung cancer the prevalence of *EGFR* mutations varies from 10% in Caucasians to more than 40% in Asian populations [6]. They are mainly located in the tyrosine kinase domain and 90% consist of either small deletions in exon 19 (DEL19) or a missense mutation in codon 21 that changes the leucine 858 in an arginine (p.L858R). Concerning rare alterations, about 3% of the mutations occur at codon 719, resulting in the substitution of glycine by a cysteine, alanine or serine (p.G719X) or at codon 861 (p.L861Q) [7,8]. In addition, there are rare 1 to 2% in-frame insertion mutations in exon 20 [9]. The predictive value of frequent alterations (DEL19 and p.L858R) is more or less equivalent but some studies have reported a higher sensitivity and longer PFS for patients with DEL19 mutated NSCLC [10]. Concerning rare alterations, sensitivity to EFGR-TKI and PFS are globally lower [11]. In 2010, results from large phase III trial led to the restriction of EGFR tyrosine kinase inhibitors to *EGFR* mutated tumors in first line treatment [12]. *EGFR* mutational status has therefore became mandatory to determine which therapy will be the most appropriate to patients with stage IV diseases. In this context, genetic heterogeneity is an obstacle to correct determination of *EGFR* status on small biopsies specimens. Previous studies showed that the *EGFR* mutation status was discordant in different parts of the tumor or between primary or secondary metastatic sites [13-16]. At the opposite, Yatabe et al showed in a series of Asiatic patients that discordant cases where extremely rare [17] and it was suggested that discrepancies regarding *EGFR* mutations distribution could be due to methodological procedures [16-18]. If *EGFR* genotyping results depend on sample types it will negatively impact treatment decisions. In Caucasians, *EGFR* molecular status at various tumor sites remains to be examined in standard testing conditions to validate *EGFR* molecular testing as a diagnostic tool. Finally, *EGFR* mutational heterogeneity could also explain the occurrence of secondary EGFR-TKI resistances. Patients undergoing EFGR-TKI treatments will ultimately relapse. Recurrences are related to various mechanisms among which the emergence of *EGFR* p.T790M clones seems to be the most frequent. This alteration, present as a minor sub-clone before treatment seems selected by EGFR-TKI treatments [19]. Most methods used for molecular diagnostic are not sensitive enough to detect minor p.T790M subclones (<1%) and this alteration is rarely identified in untreated patients. Indeed, the reported frequency of baseline *EGFR* p.T790M mutations varies widely in the literature, ranging from 1% of all *EGFR*-mutant lung cancers [11] to 35% of all *EGFR*-mutant lung cancers [20] and depends of the sensitivity of the assays used and their ability to identify minor clones within a tumor. The prognostic significance of baseline *EGFR* p.T790M has not been reported. In the acquired resistance setting, it has been demonstrated that the presence of p.T790M predicts a favorable prognosis and indolent progression, compared to the absence of p.T790M after TKI failure [19].

Because no large Caucasian series was tested for *EGFR* genetic heterogeneity, we addressed this question in clinical testing conditions thanks to a French nationwide *EGFR* mutation characterization program in advanced lung cancer (National Cancer Institute, INCa).

The aim of this study was to answer several questions of clinical relevance: -i- is *EGFR* mutation distribution, including p.T790M, heterogeneous within the primary tumor?; -ii- is *EGFR* mutation distribution heterogeneous between the primary tumor and thoracic metastases ?; -iii- Are microbiopsy or cytology samples suitable for EGFR screening? and iiii- is *EGFR* copy number heterogeneous within the tumor?

## Patients and methods

### Patients

We studied 357 consecutive patients with adenocarcinoma that had *EGFR* testing for clinical purpose at the Hôtel Dieu Hospital from January 2010 to June 2011 in accordance with the directive of the European Medicines Agency (stage IV NSCLC tested before EGFR-TKI treatment). We found that 57 patients out of 357 had *EGFR* mutated tumors (15.9% of the entire series). For 40 patients we had enough tissues available for a multi-localization screening. No concomitant *KRAS* or *HER2* alteration was identified on those samples. To rule out possible genotyping errors at time of diagnosis, we also tested 10 *EGFR*, *KRAS* and *HER2* wild type (WT) adenocarcinomas. This study was reviewed and approved by the local ethic committee (Comité de Protection des Personnes/CPP 2012 06-12). Patients’ characteristics are shown in Table 1 and are in accordance with previous reports on *EGFR* mutated Caucasian series [21,22].

**Table 1:** **Clinical characteristics of patients with** ***EGFR*** **mutated lung adenocarcinoma**

**Gender**	
Male	13
Female	27
**Age(years)**	Mean 64 [42 – 81]
**Tobacco status**	
≤ 10 Pack per year	15
> 10 Pack per year	14
unknown	11
TNM stage at initial diagnosis*	
I	4
II	4
III	9
IV	20
Unknown	3

Shows the clinical characteristics of patients with an *EGFR* mutated adenocarcinoma. Patients mentioned with stage 1 or 2 tumors have relapsed. *EGFR* testing was done using the available material (diagnostic samples) at time of relapse.

### Samples

All *EGFR* mutated adenocarcinomas had thyroid transcriptional factor (TTF1) nuclear expression (Novocastra, clone SPT24). The 22 surgical resections were classified according to the IASLC/ATS/ERS recommendation [23].

A total of 153 tumor fragments from 40 patients were selected by two pathologists (MLA and DD): 5 bronchial biopsies, 2 bronchial aspirations, 5 tomography-guided needle lung biopsies, 8 pleural effusions, 33 metastatic pleural localizations, 83 pulmonary surgical samples (lobectomy and wedge resections) and 17 lymph-nodes specimens. For each patient 2 to 19 loci were generated consisting of different localizations (primary and metastatic sites) or subdivision of the tumor tissue into small parts to analyze *EGFR* mutation at different sites, with different tumor cell content and different architectural patterns (Table 2, Additional file 1: Table S1). We called “small specimens”: cytology (pleural effusions or bronchial aspiration) or small biopsy (tomography-guided needle lung biopsies or bronchial biopsies). Concerning wild type samples 39 fragments from 10 patients were generated, including 3 adrenal glands, 6 lymph-nodes, 2 pleural metastases and 28 fragments from the primary tumors. All contained more than 50% tumor cells to rule out possible mutant allele dilution (Additional file 2: Table S2).

**Table 2:** **Multi**-**localization** ***EGFR*** **genotyping screening**

	**Number and type of samples analyzed per patients**					**EGFR genotyping results**			
**Patients**	**Mutation type**	Primary tumor(sugical sample,lung biopsy*,bronchial biospy£)	**PLEURAL BIOPSY**	**LYMPH NODE**	Cytology: Pleural Effusion,Bronchial aspiration*	**Contributive samples**	**Concordant EGFR status**	**Non contributive samples**	**Discordant EGFR status**
P1	del19	7	0	0	0	7	7	0	
P2	del19	2	0	0	0	2	2	0	
P3	del19	0	3	0	*1*	4	3	0	*1 (rescued by CAST PCR)*
P4	del19	0	4	0	1	5	5	0	
P5	del19	0	3	0	1	4	4	0	
P6	del19	1* + 1*	0	0	0	2	2	0	
P7	del19	0	0	0	2	2	2	0	
P8	del19	1* + 1*	0	0	0	2	2	0	
P9	del19	3	1	0	0	4	4	0	
P10	del19	0	3	0	0	3	3	0	
P11	del19	0	3	0	0	3	3	0	
P12	del19	0	3	0	0	2	2	1	
P13	del19	4	0	0	0	4	4	0	
P14	del19	2	0	0	0	2	2	0	
P15	del19	0	2	0	1	3	3	0	
P16	del19	1£ + *1£*	0	0	1*	3	1	0	*2 (remained WT by CAST PCR)*
P17	del19	3	0	0	0	3	3	0	
P18	del19	0	0	1	1*	1	1	1	
P19	del19	1 + 1*	0	0	0	2	2	0	
P20	p.G719A	1	0	4	0	5	5	0	
P21	p.G719A	5	0	2	0	7	7	0	
P22	ins20	3	0	1	0	4	4	0	
P23	ins20	0	2	0	0	2	2	0	
P24	ins20	3	0	2	0	4	4	1	
P25	ins20	0	2	0	0	2	2	0	
P26	ins20	3	0	0	0	3	3	0	
P27	ins20	2 + *1*	0	1 + *1*	0	4	2	1	2
P28	p.L858R	4	0	2	0	6	6	0	
P29	p.L858R	1£ + 1£	0	0	0	2	2	0	
P30	p.L858R	2	0	0	0	2	2	0	
P31	p.L858R	3	0	0	0	3	3	0	
P32	p.L858R	0	3	0	1	4	4	0	
P33	p.L858R	0	3	0	0	3	3	0	
P34	p.L858R	1£	1	0	1	3	3	0	
P35	p.L858R	4	0	2	0	6	6	0	
P36	p.L858R	2	0	1	0	3	3	0	
P37	p.L858R	3	0	0	0	3	3	0	
P38	p.L858R	3	0	0	0	3	3	0	
P39	p.L858R	3	0	0	0	3	3	0	
P40	p.L861Q/p.T790M	19	0	0	0	19	19	0	

Shows the type and number of samples tested for *EGFR* mutation in 40 patients. Non-contributive samples are non-amplified specimens. Discordant samples are DNAs that have been found wild type when a mutation was expected. The discordant samples are italic. Lung biopsy: *and bronchial biopsy: £.

In order to fit to the usual clinical practice of our pathology department, *EGFR* mutations were analyzed in a representative tumor area, without microdissection procedure. All samples (153 from EGFR mutated tumors, 39 from wild type samples) were reviewed for tumor cell content at x100 magnification (HES staining) by pathologists (MLA and DD) for 26 out of the 153 samples had low tumor cell contents (Additional file 1: Table S1 and Additional file 2: Table S2).

### Molecular analysis

Genomic DNA was extracted from 20 μm-thick formalin-fixed paraffin-embedded blocks using illustra™ DNA extraction kit BACC2 (GE Healthcare), according to the manufacturer's instructions. *EGFR* mutations were analyzed using locally validated tests [24]. In the diagnostic setting, our screening strategy is to test *EGFR* exon 19 deletions (DEL19), exon 20 insertions (INS20) and the p.L858R mutation along with *KRAS* and *HER2* exon 20 insertions. Non-mutated samples are subsequently analyzed for *EGFR* codons 719, 861 and mutated samples for *EGFR* p.T790M. The 153 fragments generated from mutated samples were analyzed for the mutation found at initial diagnosis, the 39 fragments from non-mutated cases were tested for the entire *EGFR* mutation panel (DEL19, INS20, p.L858R, p.L861Q and p.G719X).

Deletions and insertions were detected using fragment analysis with a FAM-labeled primer, run on an ABI 3730 XL (Applied biosystems, Foster City, CA) and analyzed with Genemapper software (Applied biosystems). Fragment analysis has a detection cut-off of 5-10% mutated/wild type allele ratio. Samples with an expected DEL19 that were found wild-type (n = 3) using fragment analysis were subsequently re-analyzed by TaqMan® Mutation Detection assays based on Competitive Allele Specific TaqMan PCR technology (CAST). Point mutations: p.L861Q, p.G719A, p.G719C, p.G719S, p.L858R and p.T790M were analyzed using similar technology. CAST probes were not available to test INS20 mutations. Allele specific assays were run in a final volume of 5 μl in 384 wells plate including 2.5 μl of 2X TaqMan® genotyping master mix (Applied Biosystems), 0.5 μl of 10X Assay Mix (Hs00000173_rf :EGFR_rf; Hs00000141_mu :EGFR_6213_mu; Hs00000104_mu: EGFR_ 6239_mu; Hs00000148_mu: EGFR_6253_mu; Hs00000146_mu: EGFR_6252_mu; Hs00000106_mu: EGFR_6240_mu; Hs00000102_mu: EGFR_6224_mu; Hs00000228_mu: EGFRex19dels_mu from Lifetechnologies) and 1μl DNA template (≤20ng/μl). Runs were performed in duplicates on an ABI Prism 7900 HT sequence detection system (Applied Biosystems) using the following thermo cycling conditions: 95°C/10 m (92°C/15 s, 58°C/1 m) for 5 cycles, then (92°C/15 s, 60°C/60 s) for 40 cycles and analyzed with the SDS 2.0 software program (Applied Biosystems). qPCR analyses have a detection cut-off of 1-2% mutated/wild type allele ratio [24]. Direct sequencing was run on a subset of samples as previously described [25].

### EGFR copy number

EGFR copy number was assessed by real time quantitative PCR using TaqMan® copy number assays. Three probes were selected, in intron1, on intron7-exon8 and exon29-intron29 boundaries (Hs04960197_cn; Hs01646307_cn; Hs00458616_cn). TaqMan® Copy number reference assay RNase P was used as internal control and calibration was done using non-tumor formalin fixed paraffin embedded (FFPE) tissues. Samples were run in triplicates, the maximum difference tolerated between triplicates to calculate the average cycle threshold (Ct) was 0.3. *EGFR* copy number was given by the formula 2^-ddct^ as described previously [26]. Increased copy number was defined by a CNV > 2.5. Cell lines with known copy number variations (CNV) were used as controls. FFPE samples with RNase P Ct over 30 were not used for copy number quantification and qualified as non-contributive samples.

## Results

1- *EGFR* mutation status on multiple spatially separated samples:

From January 2010 to March 2011, 357 patients managed at the Hotel-Dieu hospital for lung cancer had *EGFR* testing for clinical purpose. Among the 57 *EGFR* mutated tumors, 40 patients had enough available tissue for multiple *EGFR* testing. Nineteen patients had an exon 19 deletion (DEL19), 12 a p.L858R point mutation, 6 an exon 20 insertion (INS20), 2 a p.G719A mutation and 1 a p.L861Q/p.T790M double alteration. For those 40 patients, 153 DNA samples were extracted from various tumor localizations and reanalyzed for the initial alteration. Four samples could not be amplified (2 DEL19 and 2 INS20 tumors). The initial *EGFR* mutation was identified in 144 of the 149 informative samples. Five samples with an expected DEL19 (n = 3) or INS20 (n = 2) were found wild type. For those 5 cases, 2 cytologic specimens (pleural effusion and bronchial aspiration), 1 small endobronchial biopsy and 2 surgical specimens, the proportion of tumor cells was low, ranging from 2% to less than 10%. One specimen (P3) was rescued by DEL19-CAST probes while both samples from P16 remained wild type (Table 2). CAST probes were not available to test INS20. There was no discordance for samples with expected p.L858R, p.L861Q/p.T790M or p.G719A mutations (Table 2 and Additional file 1: Table S1).

All specimens were tested for the p.T790M mutation. This alteration was identified in one tumor (P40). Patient was naïve of EGFR-TKI, the tumor was p.L861Q/p.T790M mutated, was divided into 19 parts and both mutations were homogeneously distributed with similar allele intensity (Additional file 1: Table S1). Samples that were initially diagnosed *EGFR* wild-type, were found wild-type on all sub-specimens (Additional file 2: Table S2).

2- Concordant *EGFR* mutation status was found within the tumor, and between primary and thoracic metastasis.

In our series, 22/40 patients had surgical resection for adenocarcinoma allowing classification according the IASLC/ATS/ERS recommendations [2]. All architectural patterns were represented except the mucinous pattern. Most tumors had a lepidic counterpart (9/22) but it was not necessary the predominant pattern (Additional file 1: Table S1). For these 22 adenocarcinomas, we analyzed different tumor area, displaying similar or different architectural patterns and no *EGFR* heterogeneity was seen within these specimens.

For 10 patients we analyzed both primary tumor and lymph node metastases (n = 8) or pleural metastases (n = 2). A single discordant result was found between the primary tumor (INS20 mutation) and one metastatic lymph node containing 15% of tumor cells. For this patient, the mutation was present in the other lymph node metastasis (P27, Table 2, Additional file 1: Table S1).

Two patients showed a pre-invasive lesion (atypical adenomatous hyperplasia and in situ adenocarcinoma) located at distance of the invasive adenocarcinoma. These pre-invasion lesions had the same *EGFR* mutation as the invasive counterpart (Additional file 1: Table S1).

3- Microbiopsy and cytology samples allow *EGFR* mutation analysis.

Twenty non-surgical specimens (8 pleural effusions, 2 bronchial aspirations, 5 tomography-guided needle lung biopsies, 5 bronchial biopsies) were analyzed including 8 with less than 15% tumor cells. Nineteen samples were contributive, 16 were *EGFR* mutated and 3 were wild type: 1 pleural effusion, 1 bronchial aspirate and 1 bronchial biopsy with 5, 5 and 10% tumor cell content respectively (Table 2).

4- Determination of *EGFR* copy number.

*EGFR* copy number (CN) was available in 132/153 samples. In this series, 26/132 samples had an *EGFR* CN > 2.5 and 2 an *EGFR* CN >5. Half of the patients (18/40) had at least one sample showing an increased *EGFR* CN but only one patient had homogeneous copy number increase on all fragment analyzed (CN >4 on 3 pleural biopsy fragments, P11) (Table 3).

**Table 3:** **Multi**-**localization** ***EGFR*** **CNV screening**

	**Number and type of samples analyzed per patients**					***EGFR*** **CNV results**		
**Patients**	**Mutation type**	Primary tumor(sugical sample,lung biopsy*, bronchial biospy£	**Pleural biopsy**	**Lymph node**	Cytology: Pleural effusion,Bronchial Aspiration*	**EGFR CNV ≥ 2.5**	**EGFR CNV** < **2.5**	**Non contributive samples**
**P1**	**del19**	7	0	0	0	5	2	
**P2**	**del19**	2	0	0	0		2	
**P3**	**del19**	0	3	0	1		3	1
**P4**	**del19**	0	4	0	1	1	3	1
**P5**	**del19**	0	3	0	1	1	3	
**P6**	**del19**	1* + 1*	0	0	0	1	1	
**P7**	**del19**	0	0	0	2	1	1	
**P8**	**del19**	1* + 1*	0	0	0	1	1	
**P9**	**del19**	3	1	0	0	1	3	
**P10**	**del19**	0	3	0	0		1	2
**P11**	**del19**	0	3	0	0	3		
**P12**	**del19**	0	3	0	0	1	2	
**P13**	**del19**	4	0	0	0		4	
**P14**	**del19**	2	0	0	0		2	
**P15**	**del19**	0	2	0	1		3	
**P16**	**del19**	1£ + 1£	0	0	1*		1	2
**P17**	**del19**	3	0	0	0	1	1	1
**P18**	**del19**	0	0	1	1*		1	1
**P19**	**del19**	1 + 1*	0	0	0		1	1
**P20**	**p.G719A**	1	0	4	0	1	2	2
**P21**	**p.G719A**	5	0	2	0		6	1
**P22**	**ins20**	3	0	1	0	2	2	
**P23**	**ins20**	0	2	0	0		2	
**P24**	**ins20**	3	0	2	0		4	1
**P25**	**ins20**	0	2	0	0	1	1	
**P26**	**ins20**	3	0	0	0		3	
**P27**	**ins20**	2 + 1	0	1 + 1	0		4	1
**P28**	**p.L858R**	4	0	2	0		6	
**P29**	**p.L858R**	1£ + 1£	0	0	0	1	1	
**P30**	**p.L858R**	2	0	0	0		2	
**P31**	**p.L858R**	3	0	0	0		3	
**P32**	**p.L858R**	0	3	0	1	2	2	
**P33**	**p.L858R**	0	3	0	0	1	1	1
**P34**	**p.L858R**	1£	1	0	1	1		2
**P35**	**p.L858R**	4	0	2	0		6	
**P36**	**p.L858R**	2	0	1	0		2	1
**P37**	**p.L858R**	3	0	0	0		2	1
**P38**	**p.L858R**	3	0	0	0	1	2	
**P39**	**p.L858R**	3	0	0	0		2	1
**P40**	**p.L861Q/p.T790M**	19	0	0	0		18	1

Shows the type and number of samples tested for *EGFR* copy number variation (CNV) in 40 patients. Non-contributive samples are non-amplified specimens or samples with Ct >30.

Lung biopsy: * and bronchial biopsy: £.

Concerning specimens with very low tumor cell content (≤10%, n = 25), 5 were found wild type and 2 were non informative, none of these 7 specimens had an increased *EGFR* CN. At the opposite, among the mutated specimens with low tumor cell content 4 /18 had a CN ≥ 2.5 suggesting that *EGFR* CN impacts on mutation detection for low tumor cell content specimens.

**Figure 1:**
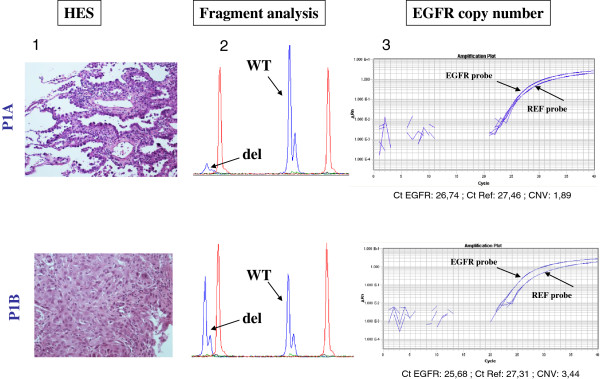
**Illustration of the different patterns of *EGFR *DEL19 mutation, *EGFR *CN analyses and the associated histological features for patient P1.** -**1**- HES staining at x100 magnification, P1A : lepidic pattern, P1B: solid pattern. -**2**- Fragment analysis for EGFR DEL19 mutation. -**3**- EGFR copy number evaluation. In sample A with lepidic pattern, mutation was validated by a cast PCR assay and there is no increased copy number of *EGFR* gene (CNV:1.89). In sample B with solid pattern, *EGFR* amplification was identified (CNV: 3.44).

We next examined whether increased *EGFR* CN was associated with specific morphologic features. In our experience, there was no clear association with high-grade lesions (solid or micropapillary predominant patterns) but in one patient, high *EGFR* CN was found in the solid pattern and not in the lepidic counterpart (Figure 1). At the opposite, 1 out of the *EGFR* mutated precursor lesions (in situ adenocarcinoma and atypical adenomatous hyperplasia), had an increased *EGFR* CN (CN: 3.4). No *EGFR* copy number increase was found in *EGFR* wild type tumor samples.

## Discussion

Tyrosine kinase inhibitors (TKI) have changed advanced *EGFR* mutated lung carcinoma clinical practice with global improved short-term survival and fewer side effects [27]. In routine clinical practice *EGFR* mutation screening is mandatory to decide which first line treatment would be the most appropriate [28,29]. Heterogeneous repartition of *EGFR* mutations within tissue or between different metastatic sites is an obstacle to accurate molecular screening. It was reported in different studies and remains an important question for clinicians [13-15,30-32]. Table 4 summarizes previous works and compared samples and technologies. Main differences are, the inclusion of various cancer types including squamous-cell cancers that are not targets for EGFR-TKI treatments or pre-treated samples, different proportion of smokers, different types of metastatic site and finally different technologies and different panels of mutations tested. It seems that higher proportion of smokers, heterogeneous tumors specimens and the use of low sensitivity methods yielded to higher rates of inconsistencies in *EGFR* mutation results [33,34]. Moreover lower rates of concordance are also found for rare *EGFR* variants [34,35]. It seems therefore important to analyze in a series of patients prospectively tested for *EGFR* mutation in clinical settings, the impact of heterogeneity on diagnosis. Indeed, non-surgical specimens from either the primary or metastatic site often constitute the only tissue available for molecular diagnosis in patients eligible for TKI with advanced stage cancers [13,30,32,36,37]. Are these small specimens reliable for molecular testing? We addressed this question in Caucasian patients with *EGFR* mutated lung tumors. Multiple samples were obtained either from different localizations or different loci within the primary tumor. As expected in Caucasians, the frequency of *EGFR* mutated tumor was 15% [38]. The p.T790M resistance mutation was studied in all patients (153 specimens) that were EGFR-TKI naïve. In untreated patient, the p.T790M mutation is usually described as a subclonal alteration that is difficult to identify even with highly sensitive methods [39]. Here the alteration was found once concomitantly to a p.L861Q mutation, both alleles were equally represented and the distribution was homogeneous suggesting that in this case, the p.T790M alteration is a driver mutation altogether with the p.L861Q. Routine *EGFR* testing gives a qualitative analysis of *EGFR* mutations, tumor is either mutated or non-mutated and the proportion of mutated allele is not taken into consideration. Using this definition, we found that 149/153 samples were contributive; among these 149 samples, only 5 showed a discordant genotype (WT/M). Discordant results were from low tumor cell content specimens expected to be either DEL19 or INS20. Fragment analysis, the method used to detect these alterations has a higher detection threshold as compared to allelic discrimination (10% versus 1%) [24], suggesting that these results might be false negative. Among the samples that could be tested using the DEL19 TaqMan® Mutation Detection assay one was found positive while both specimens from patient P16 remained negative. This patient had 2 primary cancers, concomitant adenocarcinoma and squamous cell cancer. Initial *EGFR* mutation was found on a bronchial biopsy sample showing an ADC, a second biopsy and the bronchial aspiration were found *EGFR* wild type even though high sensitivity CAST PCR was used. For this patient, the existence of 2 cancers might explain the presence of *EGFR* mutated and non-mutated samples. Indeed diagnosis on small specimens may be equivocal. To validate the accuracy of *EGFR* wild type status, 10 patients with a diagnosis of *EGFR* wild type tumor were re-analyzed at different loci. No *EGFR* alteration was found (mutation or increased CN). It suggests that negative results can be trusted as long as the method detection cut-off matches the tumor cell content. Although our work cannot rule out the existence of minor wild type subclones, we believe that any sample allows accurate *EGFR* mutations detection at initial diagnostic. This fits the results of Yatabe et al [17]. However, discrepancies may be increased by the use of low sensitivity methods such as sequencing. In this work, 38 samples were analyzed by direct sequencing. We found 23 concordances, 3 discordances and 12 informative sequences because of high background noise. We stopped the comparison as this method is not used in diagnosis in our lab and was shown to be inappropriate for biopsy or cytology FFPE samples. Indeed results depend on the estimation of the tumor cell content, on the method’s detection cut-off and on EGFR copy number. What threshold of tumor nuclei should we use? It is assumed that more than 50% of tumor cells allows accurate molecular testing and that samples under 10% may lead to false negative results [40]. Between 10% and 50% the reliability depends on the laboratory experience. Interestingly, we had detectable *EGFR* mutations in 18/23 contributive samples with ≤ 10% of tumor cells. It highlights the fact that if a positive result can be found in a sample with low tumor content, negative results have to be validated according to the specimen’s tumor content. Pleural metastatic evolution is frequent in lung cancer and pleural effusion and/or tissue are convenient materials for molecular biology.

**Table 4:** Summary of previously published series

**Article**	**Histological subtype**	**No of samples**	**Smoking status**	**Concordance PT/M**	**Analysis within PT**	**Concordance within PT**	**Method**	**Mutation type**	**Metastatic sites**
Yatabe et al. 2011 [17]	ADC (77)	77		100			qPCR	L858R	Lymph nodes
							Fragment analysis	DEL19	
								INS20	
								719X	
Sun et al. 2011 [37]	ADC (39), SCC (31), ADSQ (6), LCC (4)	80	Ever (49)				Direct sequencing	all	Lymph nodes
			Never (31)						
			Global	92,5% (74/80)					
Wei et al. 2014 [41]	ADC (49)	50	Ever (10)	80%			qPCR (commercial kit)	45 hotspots	Lymph nodes
	SCC (1)								
			Never (40)	97,5%					
			Global	93% (47/50)					
				
Bai et al. 2013 [36]	ADC (63)	85 (45 EGFRmt 40 EGFRwt)			1431 foci	87,1%	ARMS	DXS EGFR mutation Kit	
	SCC (10)				1238 foci (foci : capture with laser microdissection 0,1cm2)	4 cases with 5% - 8% of foci showing mutations			
	ADSQ (5)								
	Other (7)								

Chang et al. 2011 [42]	ADC (34)	56 (27 EGFRmt)	Ever (29)	62%			Direct sequencing	all	Lymph nodes
	SCC (17)								
	ADSQ (1)		Never (23)	70%					
	Other (1)		Unknown (4)						
			Global	68% (38/56)					
Schmid et al. 2009 [33]	ADC (96)	96 (7 EGFRmt)	Ever (74)				Direct sequencing	L858R (3)	Lymph nodes
								DEL19 (3)	
			Never (22)					INS20 (1)	
			Global	14% (1/7)					
Gow et al 2009 [34]	ADC (42)	67 (35 EGFRmt)	Ever (26)				Direct sequencing and ARMS for discordant results	all	Brain (25)
	SCC (21)								Bone (20)
	ADSQ (0)	(19 with adjuvant treatment before molecular analysis on metastatic site)	Never (41)						Other (22)
	Other (4)		Global	26% (9/35) seq and 57 %(20/35) ARMS					
Mattsson et al. 2012 [18]	ADC (6)	6			3 foci per tumor (distinct morphologies)	100%	Direct sequencing	L858R and DEL19	
									
Kalikaki et al. 2008 [35]	ADC (20)	25 (7 EGFRmt)	Ever (22)				Direct sequencing	all	Brain (3)
	SCC (2)								Pleura (5)
	ADSQ (0)	(17 with adjuvant treatment before molecular analysis on metastatic site)	Never (3)						Lung (9)
	Other (3)		Global	14% (1/7) 5 mutations are rare alterations (codons 692-847-746-857)					Adrenal gland (3)

									Bone (2)
									Skin (1)
									Liver (1)
Matsumoto et al. 2006 [43]	ADC (19)	19 (12 EGFRmut)		100%			Direct sequencing	L858R, DEL19	Brain (19)
Yatabe et al. 2011 [17]	ADC (50)	50 EGFRmt			3 foci per tumor (50)	100%	qPCR	L858R	
							Fragment analysis	DEL19	
					100 foci per tumor (5)	100%			

Schematic review of previously published series comparing primary tumor and metastasis or different loci within primary tumor. Tumor type, smoking status, detection methods, mutation tested and metastatic sites are given. PT : primary Tumor, M: metastasis, ADC: adenocarcinoma, SCC: squamous cell carcinoma, ADSQ : adenosquamous carcinoma, LCC: large cell carcinoma mt: mutated, wt : wild type, ARMS: amplification refractory mutation system.

We secondly tested whether *EGFR* CN increase could explain why *EGFR* mutation assessment, remain possible for cases with very few tumor cells. We found that *EGFR* copy number was heterogeneous between different fragments from the same tumor. Thus, the allelic ratio of each specimen depends not only on the tumor cell content but also on the *EGFR* copy number and on the number of mutated allele in tumor cells. In clinical settings the proportion of *EGFR* mutated cells or the presence of an associated increased copy number, which is frequent in *EGFR* mutated tumors, is not taken into account. In samples with various tumor cell contents and various *EGFR* gene copy number the quantification of the mutated allele/wild type allele ratio is difficult. It is clear to us that this ratio varies between tumor sites and that low tumor cell content specimens are rescued by the presence of a high mutant/wild type allele ratio. At the opposite, high tumor cell content specimens may show low mutant/wild type allele ratio estimated by the intensity of the mutant probe signal. This variability may explain discrepancies between series using different detection strategies or using microdissection of very few tumor cells as PCR amplifications in those conditions may lead to amplification errors [44]. However, it would be important to set up assays to analyze the allelic ratio’s impact on response to treatment. One other question was the link between histology and *EGFR* mutation or CN. In our experience, no difference in *EGFR* mutation status was found according to adenocarcinoma architectural patterns. This is in accordance with a report on a small series of samples (n = 11) from a Swedish group [18]. As already reported *EGFR* copy number was variable within the same tumor and may vary according to the architectural pattern [17,45]. Here, for one tumor, *EGFR* increased copy number was restricted to the solid counterpart as compared to lepidic pattern. But this could not be generalized as other solid or micropapillary predominant pattern tumors had no EGFR CN increase and at the opposite a preinvasive lesion showed a high *EGFR* copy number. Finally, our results suggested that the role of *EGFR* amplification in cancer progression might not be directly linked to the histological tumor grade.

Patients undergoing EGFR TKI treatment will develop resistance after several months. Heterogeneity has been described as a phenomenon that could drive secondary resistance. Issues are either the development of a p.T790M or a wild type subclone [15,30,39]. In our series, there was no p.T790M heterogeneity. Considering that our detection method (CAST-PCR) can detect approximately 1 to 2% of mutated alleles in a background of wild type this might not have been sufficient for p.T790M subclone detection [24]. It was also suggested that secondary resistances might be due to *EGFR* mutation loss. Our study was not designed to detect the presence of *EGFR* wild type subclones however, if this wild type tumor cell population exists, it is not an obstacle to initial *EGFR* molecular diagnosis defined by the qualitative presence of the *EGFR* mutation.

## Conclusions

This retrospective series reports multiple *EGFR* testing in lung cancer in routine diagnostic conditions and validates that molecular testing from single tumor-biopsy sample before first line EGFR-TKI may be conducted on any specimens. This is an important result for clinical practice, it indicates that *EGFR* testing is relevant on biopsies, cytological samples, lymph nodes and metastatic sites using standardized and validated procedures with a defined detection cut-off. This existence of multiple primary tumors with possible distinct genotypes needs to be considered and the development of sensitive methods is recommended because the wild type/mutated allele ratio is unpredictable. Finally, the clinical impact of the associated *EGFR* copy number increase in a subset of samples remains to be evaluated.

## Competing interest

Following authors have declared that they do not have any conflict of interests: Mansuet-Lupo A, Zouiti F, Alifano M, Charpentier MC, Ducruit V, Devez F, Lemaitre F, Damotte D and Blons H Pierre Laurent-Puig declared being a consultant or advisory role for Amgen, Roche, Merck-Serono, Genomic Health, Astellas, Sanofi, Integragen and Raindance.

## Authors’ contribution

AL carried out the molecular genetic studies, carried out tumor classification according to the IASLC/ATS/ERS recommendation, analyzed the data, and drafted the manuscript. FZ, FL carried out the molecular genetic studies, analyzed molecular data. MA recruited the patients, drafted the manuscript. CMC carried out tumor classification according to the IASLC/ATS/ERS recommendation. DV, DF carried out the pathology laboratory work. DM, PLP participated in the design of the study, performed analysis and helped to draft the manuscript. HB conceived of the study participated in its design and coordination and helped to draft the manuscript. All authors read and approved the final manuscript.

## Supplementary Material

Additional file 1: Table S1 Detailed results showing multilocalisation screening for EGFR mutation and EGFR CNV. Click here for file

Additional file 2: Table S2 Wild type sample results. Click here for file
